# Increased CpG methylation at the *CDH1* locus in inflamed ileal mucosa of patients with Crohn disease

**DOI:** 10.1186/s13148-024-01631-z

**Published:** 2024-02-14

**Authors:** Charles de Ponthaud, Solafah Abdalla, Marie-Pierre Belot, Xiaojian Shao, Christophe Penna, Antoine Brouquet, Pierre Bougnères

**Affiliations:** 1 Department of Visceral and Digestive Surgery, Hôpital Bicêtre AP-HP, Paris Saclay University, 94276 Le Kremlin-Bicêtre Cedex, France; 2 UMR INSERM 1169 and Université Paris Saclay, Hôpital Bicêtre, 94276 Le Kremlin-Bicêtre Cedex, France; 3 Groupe d’Études sur le Diabète, l’Obésité, la Croissance, GETDOC, Hôpital Bicêtre, 94276 Le Kremlin-Bicêtre Cedex, France; 4 Digital Technologies Research Center, National Research Council Canada, Ottawa, ON K1A 0R6 Canada; 5 MIRCEN Institute, CEA Paris-Saclay/site de Fontenay-aux-Roses, Bâtiment 56 PC 103, 18 route du Panorama, BP6 92265, Fontenay-aux-Roses Cedex, France

**Keywords:** Crohn disease, CpG methylation, E-cadherin

## Abstract

**Background:**

E-cadherin, a major actor of cell adhesion in the intestinal barrier, is encoded by the *CDH1* gene associated with susceptibility to Crohn Disease (CD) and colorectal cancer. Since epigenetic mechanisms are suspected to contribute to the multifactorial pathogenesis of CD, we studied CpG methylation at the *CDH1* locus. The methylation of the CpG island (CGI) and of the 1st enhancer, two critical regulatory positions, was quantified in surgical specimens of inflamed ileal mucosa and in peripheral blood mononuclear cells (PBMC) of 21 CD patients. Sixteen patients operated on for a non-inflammatory bowel disease, although not normal controls, provided a macroscopically normal ileal mucosa and PBMC for comparison.

**Results:**

In ileal mucosa, 19/21 (90%) CD patients vs 8/16 control patients (50%) (*p* < 0.01) had a methylated *CDH1* promoter CGI. In PBMC, CD patients with methylated CGI were 11/21 (52%) vs 7/16 controls (44%), respectively. Methylation in the 1st enhancer of *CDH1* was also higher in the CD group for each of the studied CpGs and for their average value (45 ± 17% in CD patients vs 36 ± 17% in controls; *p* < 0.001). Again, methylation was comparable in PBMC. Methylation of CGI and 1st enhancer were not correlated in mucosa or PBMC.

**Conclusions:**

Methylation of several CpGs at the *CDH1* locus was increased in the inflamed ileal mucosa, not in the PBMC, of CD patients, suggesting the association of *CDH1* methylation with ileal inflammation. Longitudinal studies will explore if this increased methylation is a risk marker for colorectal cancer.

**Supplementary Information:**

The online version contains supplementary material available at 10.1186/s13148-024-01631-z.

## Introduction

While its exact pathogenesis remains unknown, CD is considered to result from a dysregulated immune response to altered microbiota or other various environmental factors in genetically predisposed patients [1]. Since CD incidence has increased in newly industrialized countries [2], the westernized environment, including diet [3–5] and changes in microbiota [6], is suspected. While environmental factors still remain hypothetical, as in most multifactorial gene-environment diseases, our knowledge of genetic susceptibility has greatly increased [7]. Indeed, genome-wide association studies (GWAS) have identified as many as 71 CD susceptibility loci [8, 9]. However, most loci contribute to CD risk with low odds ratios (< 1.15), suggesting a limited genetic contribution to CD causality [10] already demonstrated by the high rates of discordance in monozygotic twins [11].

A growing body of evidence supports the role of epigenetic mechanisms in multifactorial diseases [12] due to the dynamic interaction of epigenetics with environment [13]. A widely studied epigenetic mechanisms is CpG methylation, a mitotically heritable process able to modulate the transcription of many genes [14, 15] in blood cells and solid human tissues [16]. The earliest CpG methylation marks are programmed in two waves during embryogenesis [17, 18], then, during later fetal and postnatal life, environmental cues may modify CpG methylation marks at certain loci of our genome [19–22]. On the other side, genetic variation in *cis* or *trans* plays a major role in the establishment of methylation marks, either directly or in interaction with environmental exposures [23]. CpG methylation can therefore mediate the effects of gene–environment interactions on gene expression [24]. Indeed, the level of methylation of CpGs in promoter or enhancer regions can modulate the transcription of certain genes [15], thus can play a causal role in health and diseases [25], including inflammatory bowel disease (IBD) [26, 27]. It is important to stress that, while variation in methylation marks can contribute to disease causation it can as well be a consequence of disease or disease related changes in lifestyle or treatments.

Several studies have reported differentially methylated CpG sites in the blood cells of patients with CD ileitis [28–35]. In contrast, only three studies have examined CpG methylation in intestinal tissues of adult patients with long standing CD [36]. One of them studied samples of diseased ileal mucosa collected during surgery in 5 patients with CD [37], another studied rectal biopsies of 16 CD patients (8 with and 8 without rectal inflammation) [38], the third one studied diseased ileal mucosa from 7 CD patients with perforation or fistula [39]. In addition to these studies, distinctive CpG methylation marks associated with CD were also found in purified epithelial cells from mucosal biopsies collected from terminal ileum or ascending or sigmoid colons of 43 children newly diagnosed with CD [40]. None of the four studies cited above found that the *CDH1* locus was a differentially methylated region (DMR) in CD patients.

Several reasons, however, prompted us to study methylation specifically at the *CDH1* locus in the intestinal mucosa of CD patients. First, the *CDH1* gene encodes E-cadherin, a transmembrane glycoprotein expressed in epithelial cells and a major actor in cell adhesion, intestinal barrier, and dynamic balance of epithelial tissues [41]. E-cadherin contributes to epithelial-to-mesenchymal transition (EMT) a phenomenon that allows the conversion of adherent epithelial cells to a mesenchymal cell phenotype, which enhances migratory capacity and invasiveness [42], physiological functions that are at the forefront of the pathogenic phenomena leading to CD. In addition, fine-mapping studies of the 16q22.1 region found that rs16260, a single-nucleotide variant located in the *CDH1* promoter, is associated with ileal CD [9]. Functionality of this SNP has been demonstrated, suggesting that it could itself modulate E-cadherin expression in vivo [43–45]. Another reason for exploring methylation at the *CDH1* locus is that methylation changes at this locus have already been reported in ulcerative colitis (UC), the other major IBD [38, 46–48] and were said to contribute to the prediction of UC severity [42, 49]. If the methylation marks observed at the *CDH1* locus are triggered by intestinal UC-associated inflammation, comparable marks might also occur in response to CD-associated inflammation. In addition, *CDH1* is a tumor-suppressor gene involved in the predisposition to several cancers, notably gastric and colorectal cancer. Predisposition to colorectal cancer (CRC) occurs through both genetic predisposition [50, 51] and changes in CpG methylation [52, 53]. Since patients with CD are at increased risk of CRC diagnosis and death [54], we had an additional reason to focus on the *CDH1* locus.

The current study explored CpG methylation at the *CDH1* locus in patients with ileal CD. To find epigenetic signatures that might reflect local disease mechanisms, we measured *CDH1* methylation in inflamed mucosa. We chose a candidate gene approach focused on the *CDH1* locus instead of attempting agnostic epigenome-wide association studies (EWAS) [55, 56]. The reason was that we could not collect ileal samples in large enough numbers of patients with CD or non-IBD gut pathology to feed an EWAS, which requires analyzing several hundreds of patients and controls and is for this reason always carried out in blood cells [56, 57], rather than in the diseased tissues.

## Materials and methods

### Study population

From September 2021 to September 2022, twenty-one consecutive patients who underwent ileal or ileocolic resection in the Bicêtre Department of Digestive Surgery were prospectively included into the studied CD group. The diagnosis of CD was based on biopsies of lesions located on the colon or ileum performed during colonoscopy and enteroscopy. Crohn disease activity index (CDAI) was > 150 in all patients. Average duration of CD since diagnosis was 11.7 years (1–34 years). Imaging and endoscopic examination confirmed the presence of fistula for 12/21 and/or stricture for 14/21, leading to the surgical indication. At the time of surgery, 12/21 CD patients were treated with anti-TNFα drugs and 6/21 were receiving Azathioprine.

Sixteen patients who had non-inflammatory intestinal disease underwent ileal or ileocolic resection; they were used as a “control” group for comparison with CD patients, also they cannot be considered healthy control. This group was composed of: 4 stoma closures, 6 right sporadic colonic adenocarcinomas, 3 colonic polyps with low-grade dysplasia, 1 ileal non-inflamed stricture of undetermined etiology and 2 right colonic diverticulosis without diverticulitis. All of them had an erythrocyte sedimentation rate < 10 mm/h, normal hemoglobin and platelets, and normal C reactive protein.

The research protocol in the Department of Surgery was agreed by Paris Sud University Institutional Review Board. During the pre-operative consultation, patients signed informed consent for the current study and for genetic analysis, according to the French rules of bioethics.

Table 1 presents the main characteristics of the studied patients collected from the electronic medical record system of Bicêtre Hospital. Additional file 1: Table S1 describes the detailed results of the methylation levels for each sample analysed.

**Table 1:** Main characteristics of the studied patients

	Crohn disease N = 21	Controls N = 16
F/M	12/9	6/10
Age (years)	42 ± 19	65 ± 20
BMI (kg/m^2^)	22 ± 4	23 ± 4
Time since diagnosis (years)	12 ± 11	0.5 ± 0.5
Smokers	3	4

Controls are patients undergoing surgery for non-inflammatory intestinal diseases (see patients Section); mean (± SD); decimal values rounded to the nearest integer. The only significant difference between the 2 groups is for patient’s age (*p* < 0.001)

### Procedural methods

Venous blood samples were collected and peripheral blood mononuclear cells (PBMC) were immediately purified from fresh blood (10 mL) [58]. All CD patients had inflammatory lesions complicated by fistula (associated with intra-abdominal sepsis) and/or stricture (associated with small bowel obstruction). A surgical specimen was taken under sterile conditions in the operating room and stored for further analysis. A 1 × 1 cm sample of superficial ileal mucosa without muscularis was latter isolated from a macroscopically inflamed area and placed in a dry cryotube. The sample was frozen at − 80 °C in liquid nitrogen. For the control patients, a macroscopically healthy non-inflamed ileal specimen was taken at least 10 cm away from the surgical lesion and processed similarly.

### Choice of CpGs at CDH1 locus

We studied the CpG island (CGI) made of 104 CpGs located in *CDH1* promoter, exon 1, intron 1 and exon 2. We also selected 4 CpGs for study, named according to their position towards the transcription start site (TSS) of *CDH1* and located near 4724 bp upstream the TSS, recently identified as the 1st enhancer [59]. The FANTOM5 consortium has identified several enhancers in the *CDH1* gene using the CAGE (short for Cap Analysis of Gene Expression) technology where a bidirectional transcription CAGE pattern was detected. Particularly, the 1st active enhancer of *CDH1* chr16: 68732016-68732406 (hg38 version), was detected to interact with the promoter regions (also a CGI region) of *CDH1* https://pressto.binf.ku.dk/about.php#h_enhancers_description_long. In addition, Activity By Contact (ABC) enhancer study [60] further identified that most of them are “pleiotropic” enhancers where enhancers were detected to be contacted with multiple genes in different cell-line or tissues [61]. Additional file 1: Fig. S1 summarizes the genomic map of the *CDH1* locus with the CpG sites of interest (CGI and 4 CpGs of the 1st enhancer) and the SNP rs16260.

### DNA isolation and bisulfite conversion

Total DNA was extracted from samples and purified on spin-column with the "DNeasy Blood & Tissue kit" (Qiagen, Hilden, Germany), then stored at − 20 °C in 1.5 mL tubes. The concentrations of extracted DNA were assayed with NanoDrop spectroscopy (Nyxor Biotech, Paris, France). An optimal degree of purity and quality of the extracted DNA was assessed by 260 nm/230 nm and 260 nm/280 nm ratios. 400 ng of DNA were converted to sodium bisulfite using the EZ DNA Methylation-Gold Kit (Zymo Research Corporation, CA, USA).

### Methylation specific PCR-based bisulfite analysis

Methylation-specific PCR (MSP) requires only small quantities of DNA, is sensitive to 0.1% methylated alleles of a given CGI. Analysis of *CDH1* CGI was performed by MSP as described [62].

To amplify methylated CGI, primers were: The PCR was performed using the Taq'Ozyme HS (Ozyme, France) with a melting temperature of 52 °C. Briefly, bisulfite converted DNA were amplified using Taq'Ozyme HS polymerase (Ozyme, France) with specific primers for unmethylated or methylated DNA [respectively 5′-TTAGGTTAGAGGGTTATCGCGT-3′ (forward) and 5′-TAACTAAAAATTCACCTAC CGAC-3′ (reverse) and 5′-TAATTTTAGGTTAGAGGGTTATTGT-3′ (forward) and 5′-CACAACCA ATCAACAACACA-3′ (reverse)]. PCR mix contained 5X Buffer, 0.6 µM of each primer, 3% DMSO and 1 unit Taq DNA polymerase. Amplifications were performed in X cycles with an annealing temperature of 52 °C (methylated CGI primers) or 53 °C (unmethylated CGI primers). PCR products were assessed by electrophoretic migration on the QIAxcel Advanced automate (Qiagen, Hilden, Germany).

### Pyrosequencing-based bisulfite PCR analysis

First, a PCR amplification of the genomic sequence containing the 4 CpGs of interest was performed using unbiased primers: 5′-TTGTTATAAGGAAATTTGGAG-3′ (forward) and 5′-CCTAAAACTATACACAAACCTATC-3′ (reverse with a biotin linked in 5′ position). The PCR was performed using the Epimark Hot Start Taq DNA polymerase (New England BioLabs) with a melting temperature of 52 °C and reagents in the following proportions to give a total volume per sample of 50μL (Buffer 5X, 0.2 mM DNTP, 0.6 µM each primer, 1.25 mM MgCl2, DMSO 3% and 1.25 units / 50 µl PCR mix of Epimark polymerase).

Biotin-labeled single stranded amplicon was isolated according to protocol using the PyroMark Q96 ID Pyrosequencing instrument (Qiagen, Hilden, Germany) and underwent pyrosequencing with 0.5 μM primer. The primers used were the following according to CpG position: 5′-TGGAGTTTGTGATTTTATTA-3′, 5′-GATAGGGTTTTTTATTTAT-3′, 5′-GATGTTTGAA ATTTTATTGT-3′ and 5′-GTAATGGGTTTTATTATTT-3′. Primers (including for PCR) were generated using MethPrimer (http://www.urogene.org/cgi-bin/methprimer/methprimer.cgi) [63]. The methylation percentage for each CpG was calculated using PyroQ CpG Software (Qiagen, Hilden, Germany).

### Genotyping of SNP rs16260

The rs16260 (C > A) SNP is adjacent to the CpG Island of *CDH1*. The rs16260 SNP was genotyped by TaqMan® pre-designed SNP genotyping assay technology (LifeTech, Assay ID C_11934298_10, ThermoFisher Scientific) with TaqPath™ ProAmp Master Mix under conditions recommended by the manufacturer. PCR cycling conditions were 60 °C for 30 s, 95 °C for 5 min, 40 cycles at 95 °C for 15 s and 60 °C for 45 s. Results were generated by LightCycler® Software (Detection format: dual color hydrolysis/UPL Probe and Analysis by Endpoint Genotyping).

### Statistical analysis

Continuous data were reported as mean ± SD according to the normality of the distribution, checked graphically on a histogram and by the Shapiro–Wilk test at the 20% threshold. Intergroup comparisons of continuous variables were performed using Student t-test or the Mann–Whitney U test, depending on the distribution of the variables. Intergroup comparisons of classified variables were performed using the chi-square test or Fisher’s exact test depending on sample size. Classified variables were reported in absolute terms and percentages. The correlation between methylation of the studied enhancer CpGs was both analyzed by Pearson correlation analysis. Multivariate regressions were performed to assess the association between methylation level and Crohn disease according to the main demographic variables (sex, age, BMI). *P*-values < 0.05 were considered statistically different. All *P*-values were two-sided. The analyses were performed using R 4.1.2 for MacOS.

## Results

### Methylation of CDH1 CpG island (CGI) in mucosa and PBMC.

In the ileal mucosa, 19/21 (90%) of the CD patients had a methylated CGI of the *CDH1* promoter compared to 8/16 (50%) of the control patients (*p* < 0.01). In PBMC, 11/21 (52%) of the CD patients and 7/16 (44%) of the controls had a methylated CGI of *CDH1*. CGI methylation was more frequent in mucosa than in PBMC of CD patients (90% vs 52% in controls, *p* < 0.01), while no difference was observed between mucosa and PBMC in controls (50% vs 44% in controls, *p* = 0.7). Only 11/21 CD patients had a methylated CGI in both tissues, while 7/8 of the controls showed a methylated CGI in both mucosa and PBMC.

In the CD group, we found no association of CGI methylation in ileal mucosa or PBMC with age, sex, BMI, smoker status, treatment with anti-TNFα or azathioprine, or CDAI score. *CDH1* CGI methylation was not associated with the duration of CD. The presence of fistula or stricture was not associated with CGI methylation. In the controls, no association was found between age, sex, BMI, or smoker status and *CDH1* CGI methylation in the ileal mucosa. Three out of 6 patients with colorectal cancer had a methylated CGI in the ileal mucosa, a proportion similar to that of the entire control group.

### Methylation of CDH1 enhancer CpGs in mucosa and PBMC

**Fig. 1:**
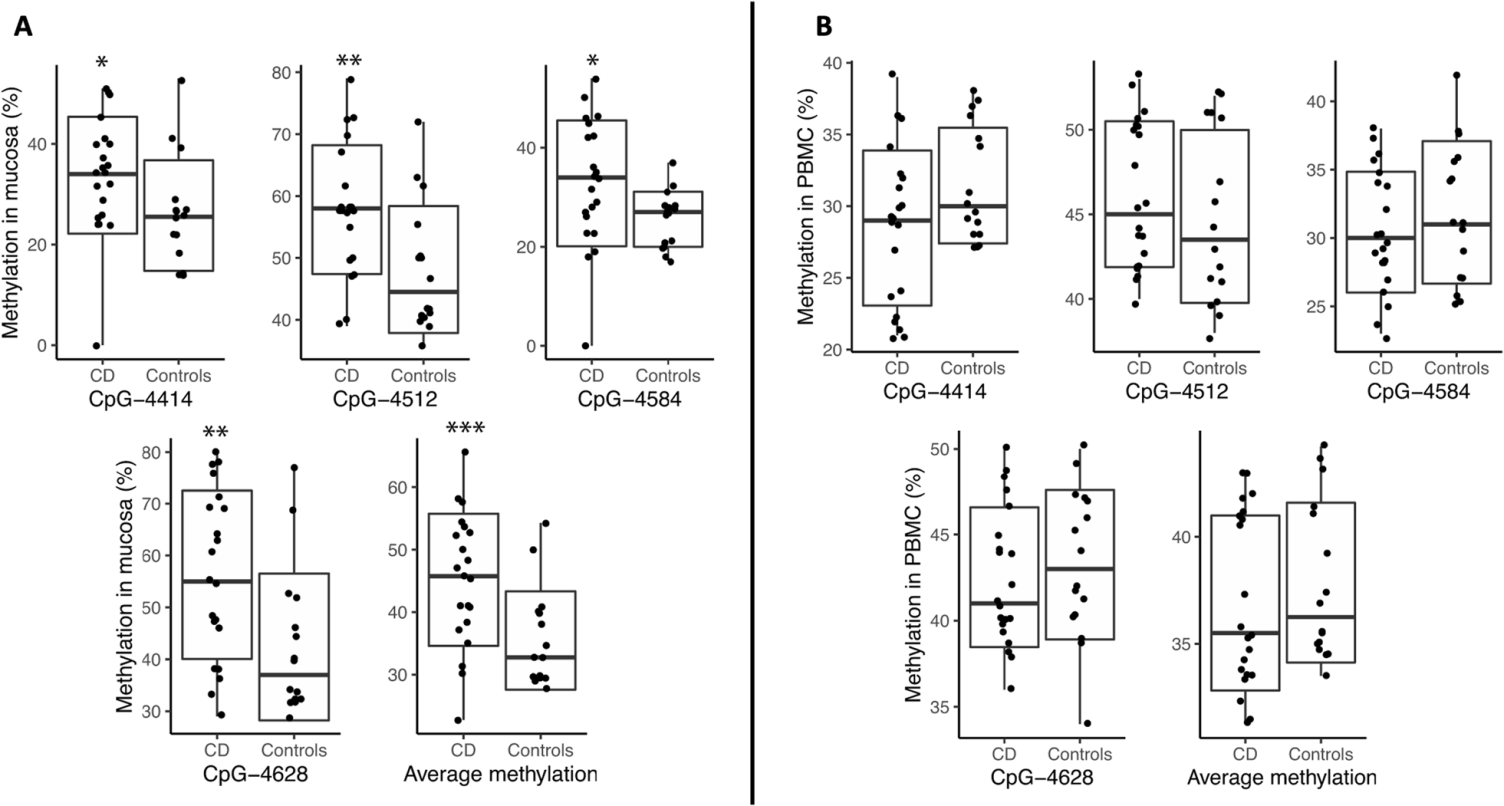
Methylation of the 4 CpGs in the 1^st^ enhancer of *CDH1* gene **A** in mucosa; and **B** in PBMC **B** patients with CD or non-inflammatory disease controls. * *p* 0.03; ** *p* < 0.02; ***p 0.01

Figure 1 describes the methylation of the 4 CpGs located in the CDH1 enhancer sequence in control and CD patients in the ileal mucosa and in circulating PBMC. Detailed results are given in Table 2. In the ileal mucosa, the degree of methylation of the 4 CpGs was significantly higher in the CD group, for each individual CpG and for the average of the 4 CpGs. A ROC curve assessing the performance of average methylation of the 4 CpGs in predicting CD yielded an AUC value of 0.85 in mucosa samples (Additional file 1: Fig S2). In contrast, *CDH1* enhancer methylation in PBMC was not different in CD and in controls. In patients with CD, the level of methylation of the 4 CpGs in mucosa was higher (45 ± 17%) than in PBMC (37 ± 9%, *p* = 0.002). In controls, no difference was observed between mucosa and PBMC.

**Table 2:** Percent methylation of the 4 CpGs of the 1st enhancer of *CDH1* gene in ileal mucosa and PBMC

	Crohn Disease *N* = *21*	Controls *N* = *16*	*P value*
Ileal mucosa:			
CpG-4414	34 ± 12	26 ± 12	0.02
CpG-4512	58 ± 11	48 ± 11	0.01
CpG-4584	33 ± 11	26 ± 11	0.03
CpG-4628	56 ± 17	42 ± 17	0.01
Average CpGs	45 ± 17	35 ± 17	< 0.001
PBMC:			
CpG-4414	29 ± 5	31 ± 4	NS
CpG-4512	46 ± 4	45 ± 5	NS
CpG-4584	30 ± 4	32 ± 4	NS
CpG-4628	43 ± 4	43 ± 4	NS
Average CpGs	37 ± 9	38 ± 4	NS

Mean (± SD); decimal values rounded to the nearest integer

There was a significant inter-correlation of methylation across enhancer CpGs in ileal mucosa (Additional file 1: Fig S3) or in PBMC (Additional file 1: Fig S4). The methylation of the 4 CpGs in ileal mucosa was not correlated with their methylation in PBMC (r = 0.3, *p* = NS). No association was found between methylation of the CGI and enhancer CpGs as shown in Additional file 1: Fig S5.

No significant association was demonstrated between methylation of enhancer CpGs and age in the control and/or the CD groups, nor with sex, BMI, or smoker status. In the CD group, no association was found with duration of clinical disease, CDAI score, medical treatment or the presence of fistula or stricture.

### Lack of association of rs16260 SNP with methylation

Additional file 1: Table S2 shows rs16260 (C > A) genotypes (no patient had an AA genotype). The rs16260 SNP was not associated with CD or with methylation of CDH1 CGI or enhancer CpGs.

## Discussion

The main finding of the current study is that both the *CDH1* promoter CGI and a group of CpGs located within the 1st enhancer of *CDH1* showed increased methylation in the inflamed ileal mucosa of CD patients. Notably *CDH1* CGI was found to be methylated in the ileal mucosa of 90% of CD patients, versus only 50% of our « control» group. Also, the increased average methylation of the 4 CpGs in *CDH1* enhancer was relatively large (45 ± 17% in CD patients vs 35 ± 17% in controls). The intestinal epithelium is constantly renewed by intestinal stem cells throughout life. It is interesting to note that the *CDH1* locus was also hypermethylated in the inflamed mucosa of patients with UC [38, 46–48] and that this methylation of the *CDH1* locus could contribute to predicting the severity of UC [33, 42, 43]. This observation supports that intestinal inflammation or its local consequences could increase methylation at the *CDH1* locus in both CD and UC. While the intestinal epithelium responds to changes in diet, age, microbiome, and immune activation, it is unknown whether these responses occur in mature or progenitor cells and whether they involve epigenetic reprogramming. Indeed, epigenetic mechanisms have recently been recognized as operating at the interface between the microbiome and the intestinal epithelial cell genome [64–66].

In PBMC, percentages of methylated *CDH1* CGI in our control group matched those observed in a study of 1036 healthy controls [67]. Consistent with previous EWAS performed in blood cells [28–35], we found no difference of CGI methylation between CD and control patients. Methylation levels of *CDH1* CGI and the 4 enhancer CpGs in PBMC correlated with those found in intestinal mucosa in our controls, as reported in normal people [68]. This correlation suggests that progenitors of PBMC and intestinal epithelium may share a common early programming of *CDH1* methylation in the endoderm germ layer, with derived cell types retaining these patterns decades later as a stable lineage mark [69]. In contrast, the absence of correlation between intestinal and PBMC methylation in CD patients supports that increased methylation has more recently occurred in progenitors or mature mucosal cells of CD patients. As discussed by Heijmans et al. [70], high inter-tissue concordance may be present for DNA methylation changes induced early in development (and potentially propagated soma-wide). In contrast, changes occurring during aging are more likely to remain tissue-specific.

Mucosal tissue from surgical specimens is composed of heterogeneous cell populations that are different in non-inflamed « control» mucosa and inflamed ileal areas of CD. There is a clear concern that our analyses, as those of others [38, 39], were conducted using whole mucosal samples containing mixed epithelial and non-epithelial cell populations, known to have different methylation [38]. Given that methylation signatures are cell-type-specific, the question arises as to whether the epigenetic patterns we observed in CD mucosa arise from the epithelial or non-epithelial cells, and whether they might be confounded by the different cell populations present in inflamed CD versus control mucosa [43]. Among non-epithelial cells, intra-epithelial lymphocytes show lineages diversity and functional states in the intestinal mucosa under both healthy and CD conditions, as well as altered spatial distribution that potentially correlates with transmural inflammation [71]. Since we did not perform mucosal cell purification [71], *CDH1* methylation could not be analyzed in infiltrated intra-mucosal lymphocytes. The only indirect information regarding mucosal lymphocytes in our CD patients comes from circulating PBMC, in which *CDH1* methylation was comparable to non-inflamed control mucosa or control PBMC. Since enterocyte number largely exceeds non-epithelial cells in inflamed ileal lesions, it is unlikely that the increased methylation levels observed in inflamed CD mucosa could be explained by infiltrated lymphocytes. Indeed, if local inflammation were able to increase CGI methylation in resident lymphocytes or non-epithelial cells, this would not result in the 90% proportion of methylated CGI that we observed in our mucosal samples.

Our findings suggest that mucosal cells of UC and CD may share epigenetic mechanisms at the *CDH1* locus despite heterogeneity in location, severity of inflammation and phenotypes [72].

The current observation may also be relevant to the risk of colorectal cancer (CRC) in CD patients. Numerous genes, notably *CDH1* have been reported to be hypermethylated and silenced in sporadic CRC [73, 74]. In addition, hypermethylation of *CDH1* CGI characterizes UC-associated CRC [53] and was supposed to serve as a useful biomarker for detecting UC patients at high risk of developing CRC [75]. Since CD increases the risk of CRC [54], the question arises whether the increased methylation that we found in CD at the *CDH1* locus could help predict disease course and associated CRC.

Clearly, methylation marks at the *CDH1* locus were not influenced by genomic sequence variation in *cis* and thus contribute to the epigenetic signature of the inflamed mucosa independently from genetics.

Many questions remain, since the current study is only observational and exploratory and carries several weaknesses. Although the ileal mucosa studied in the control group was macroscopically normal and taken out of pathological lesions, one cannot consider our control population as truly normal, i.e., non-pathological. Also, our study does not provide extensive information about all CpG residues at the *CDH1* locus. Also, it does not provide information about non-inflamed CD mucosa, as obtained from intestinal biopsies [38] in a study of CD that did not provide a comparison between non-penetrating and normal mucosa [38]. Last but not least, a major weakness of our work is the lack of information about *CDH1* expression in the ileal samples. This was due to the fact that most surgical specimens were collected and stored in conditions that would not have allowed a reliable measurement of *CDH1* mRNA.

More globally, our observation does not elucidate whether increased methylation contributes to the causality of inflammatory lesions of CD, or is instead a secondary consequence of the local inflammation induced by CD. The first hypothesis is that preexisting increased methylation predisposes some regions of the ileal mucosa of future CD patients to inflammation and possibly CRC, as it is postulated for UC. Following this hypothesis, methylation marks preexisting in the intestinal mucosa would result from epigenetic programming and environmental exposures occurring during the pre-disease life of patients. The increased methylation observed here in mucosa was not present in PBMC, and is thus posterior to the embryonic differentiation of patients’ endodermal (gut) and mesodermal (PBMC) cells. Another possibility is that the methylation changes that we observed in CD mucosa occur during post-disease life, and are induced by the local inflammation or other environmental exposures of intestinal cells, such as changes in microbiome [64–66]. In fact, the two hypotheses are not mutually exclusive. They could combine their effects to increase both the preexisting and secondary methylation at the *CDH1* regulatory locus, which might perpetuate local inflammation and trigger CRC risk in the intestinal epithelium of CD patients.

### Supplementary Information

**Additional file 1.** **Fig. S1**. Scheme of the genomic map (5’) proximal from the CDH1 gene. Studied CpGs are figured as CGI, or as lollipops for the 1st enhancer. The rs16260 A/C variant is shown.** Fig. S2**. ROC curve testing the association between CD and the methylation at the CDH1 locus in ileal mucosa. A logistic regression explored the association between CD and the enhancer CpGs according to the methylation status of CGI. The coefficient of the latter adjustment variable was used to weigh the enhancer CpGs value based on the CGI methylation status.** Fig. S3**. Correlation of the methylation of the 1st enhancer studied CpGs in the mucosa of pooled CD and control patients. R (p value).** Fig. S4**: Correlation of the methylation of the 1st enhancer studied CpGs in PBMC of pooled CD and control patients. R (p-value).** Fig. S5**. Methylation of the 1st enhancer CpGs in (A) mucosa; (B) PBMC of patients with CD or controls, according to the methylation status of the CGI. No significant differences were detected.** Table S1**. Complete individual methylation data in the studied CD patients and controls.** Table S2**. Methylation in mucosa does not depend on rs16260 (C>A) genotype. However, we observed non-significant trend for increased CGI methylation in patients with CC genotype. No had AA genotype was present. Mean (±SD); decimal values rounded to the nearest integer.

## Data Availability

The datasets used and/or analyzed during the current study are available from the corresponding author on reasonable request.
