# Correction: Increased CpG methylation at the CDH1 locus in inflamed ileal mucosa of patients with Crohn disease

**DOI:** 10.1186/s13148-024-01654-6

**Published:** 2024-03-18

**Authors:** Charles de Ponthaud, Solafah Abdalla, Marie-Pierre Belot, Xiaojian Shao, Christophe Penna, Antoine Brouquet, Pierre Bougnères

**Affiliations:** 1 Department of Visceral and Digestive Surgery, Hôpital Bicêtre AP-HP, Paris Saclay University, 94276 Le Kremlin-Bicêtre Cedex, France; 2 U1195 Inserm “Diseases and Hormones of the Nervous System” and University Paris-Saclay, 94276 Le Kremlin-Bicêtre, France; 3 Groupe d’Études sur le Diabète, l’Obésité, la Croissance, GETDOC, Hôpital Bicêtre, 94276 Le Kremlin-Bicêtre Cedex, France; 4 Therapy Design Consulting, Vincennes, France; 5 Digital Technologies Research Center, National Research Council Canada, Ottawa, ON K1A 0R6 Canada; 6 MIRCEN Institute, CEA Paris-Saclay, Fontenay-Aux-Roses, France

**Correction: Clinical Epigenetics (2024) 16:28** 10.1186/s13148-024-01631-z

Following publication of the original article [1], the authors noticed the errors in the affiliations. The second affiliation has been updated for Charles de Ponthaud, Solafah Abdalla and Pierre Bougnères. The fourth affiliation has been included for Marie-Pierre Belot. The correct affiliations and author names are given below:

Charles de Ponthaud^1,2†^, Solafah Abdalla^1,2†^, Marie-Pierre Belot^3,4^, Xiaojian Shao^5^, Christophe Penna^1^, Antoine Brouquet^1^ and Pierre Bougnères^2,3,6^*

^1^Department of Visceral and Digestive Surgery, Hôpital Bicêtre AP-HP, Paris Saclay University, 94276, Le Kremlin-Bicêtre Cedex, France

^2^U1195 Inserm "Diseases and Hormones of the Nervous System" and University Paris-Saclay, 94276, Le Kremlin-Bicêtre, France

^3^Groupe d’Études sur le Diabète, l’Obésité, la Croissance, GETDOC, Hôpital Bicêtre, 94276, Le Kremlin-Bicêtre Cedex, France

^4^Therapy Design Consulting, Vincennes

^5^Digital Technologies Research Center, National Research Council Canada, Ottawa, ON K1A 0R6, Canada

^6^MIRCEN Institute, CEA Paris-Saclay, Fontenay-aux-Roses, France.
