# Supplementary material for: Increased CpG methylation at the CDH1 locus in inflamed ileal mucosa of patients with Crohn disease
Source: Clin Epigenetics. 2024 Feb 14;16:28. doi: 10.1186/s13148-024-01631-z (PMC10865720; doi:10.1186/s13148-024-01631-z)
Supplement: Supplementary file 1 — Additional file 1. Fig. S1. Scheme of the genomic map (5’) proximal from the CDH1 gene. Studied CpGs are figured as CGI, or as lollipops for the 1st enhancer. The rs16260 A/C variant is shown. Fig. S2. ROC curve testing the association between CD and the methylation at the CDH1 locus in ileal mucosa. A logistic regression explored the association between CD and the enhancer CpGs according to the methylation status of CGI. The coefficient of the latter adjustment variable was used to weigh the enhancer CpGs value based on the CGI methylation status. Fig. S3. Correlation of the methylation of the 1st enhancer studied CpGs in the mucosa of pooled CD and control patients. R (p value). Fig. S4: Correlation of the methylation of the 1st enhancer studied CpGs in PBMC of pooled CD and control patients. R (p-value). Fig. S5. Methylation of the 1st enhancer CpGs in (A) mucosa; (B) PBMC of patients with CD or controls, according to the methylation status of the CGI. No significant differences were detected. Table S1. Complete individual methylation data in the studied CD patients and controls. Table S2. Methylation in mucosa does not depend on rs16260 (C>A) genotype. However, we observed non-significant trend for increased CGI methylation in patients with CC genotype. No had AA genotype was present. Mean (±SD); decimal values rounded to the nearest integer. [file 13148_2024_1631_MOESM1_ESM.docx]

**ADDITIONAL INFORMATION**

### Additional Figures

Suppl Fig S1: Scheme of the genomic map (5’) proximal from the *CDH1* gene. Studied CpGs are figured as CGI, or as lollipops for the 1^st^ enhancer. The rs16260 A/C variant is shown.


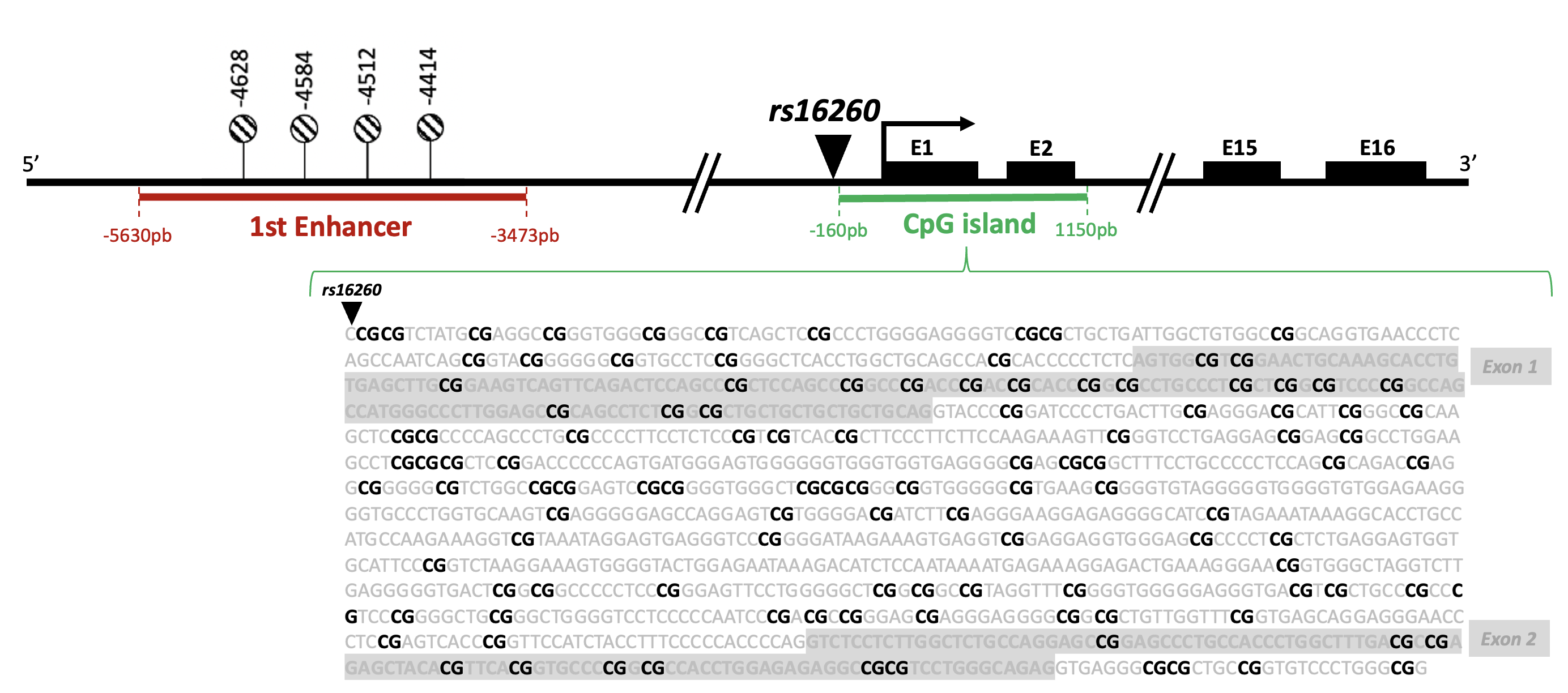


### Suppl Fig S2: ROC curve testing the association between CD and the methylation at the *CDH1* locus in ileal mucosa. A logistic regression explored the association between CD and the enhancer CpGs according to the methylation status of CGI. The coefficient of the latter adjustment variable was used to weigh the enhancer CpGs value based on the CGI methylation status.

###
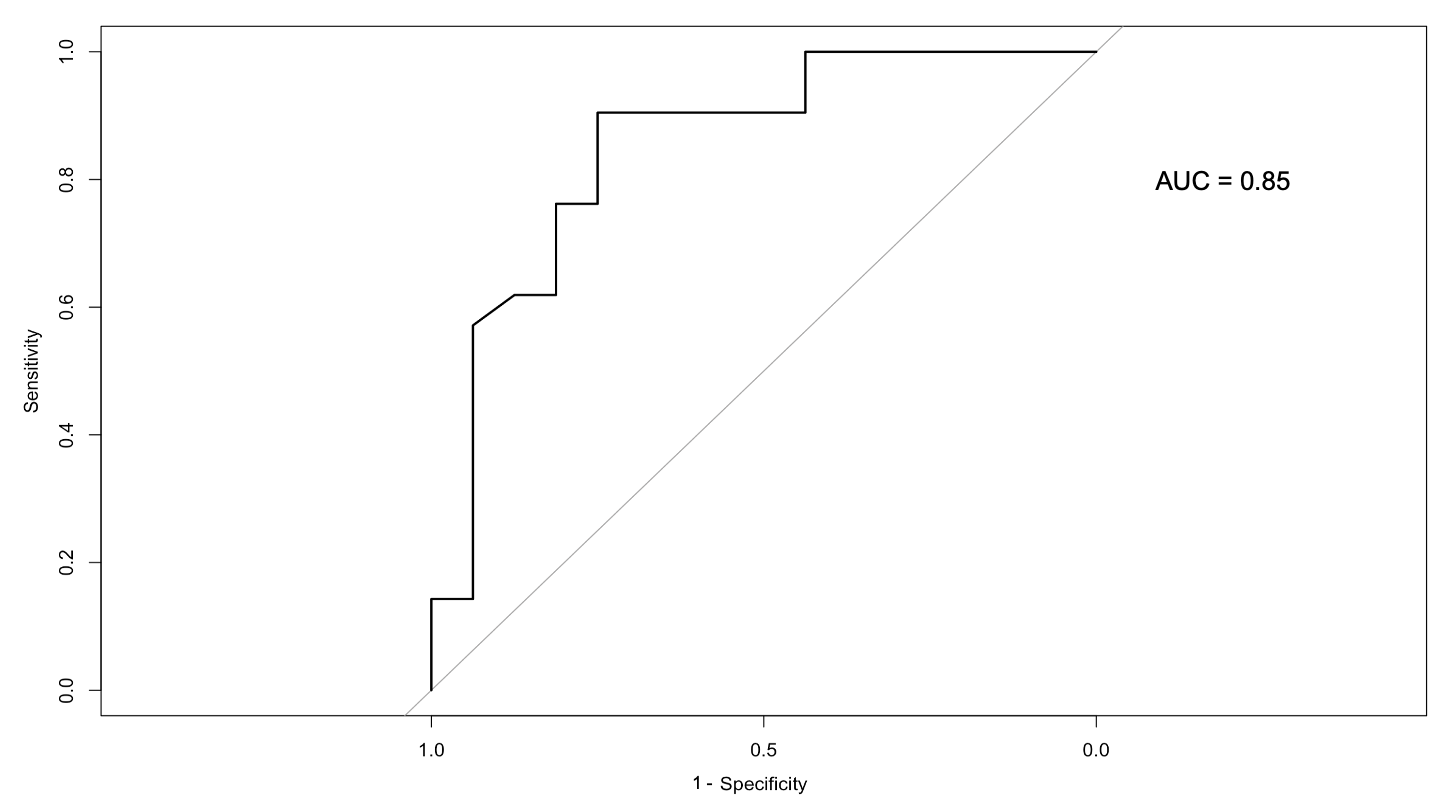


Suppl Fig S3: Correlation of the methylation of the 1^st^ enhancer studied CpGs in the mucosa of pooled CD and control patients. R (p value).

|  | **CpG**  **-4414** | **CpG**  **-4512** | **CpG**  **-4584** | **CpG**  **-4628** |
| --- | --- | --- | --- | --- |
| **CpG**  **-4414** | 1 | 0.31  (*0.060*) | 0.44  (*0.006*) | 0.52  (*0.001*) |
| **CpG**  **-4512** | / | 1 | 0.62  *(<0.001*) | 0.75  *(<0.001*) |
| **CpG**  **-4584** | / | / | 1 | 0.82  *(<0.001*) |
| **CpG**  **-4628** | / | / | / | 1 |

Suppl Fig S4: Correlation of the methylation of the 1^st^ enhancer studied CpGs in PBMC of pooled CD and control patients. R (p-value).

|  | **CpG**  **-4414** | **CpG**  **-4512** | **CpG**  **-4584** | **CpG**  **-4628** |
| --- | --- | --- | --- | --- |
| **CpG**  **-4414** | 1 | 0.67  *(<0.001)* | 0.49  *(0.002)* | 0.56  *(<0.001)* |
| **CpG**  **-4512** | / | 1 | 0.67  *(<0.001)* | 0.72  *(<0.001)* |
| **CpG**  **-4584** | / | / | 1 | 0.58  *(<0.001*) |
| **CpG**  **-4628** | / | / | / | 1 |

Suppl Fig S5: Methylation of the 1^st^ enhancer CpGs in (A) mucosa; (B) PBMC of patients with CD or controls, according to the methylation status of the CGI. No significant differences were detected.


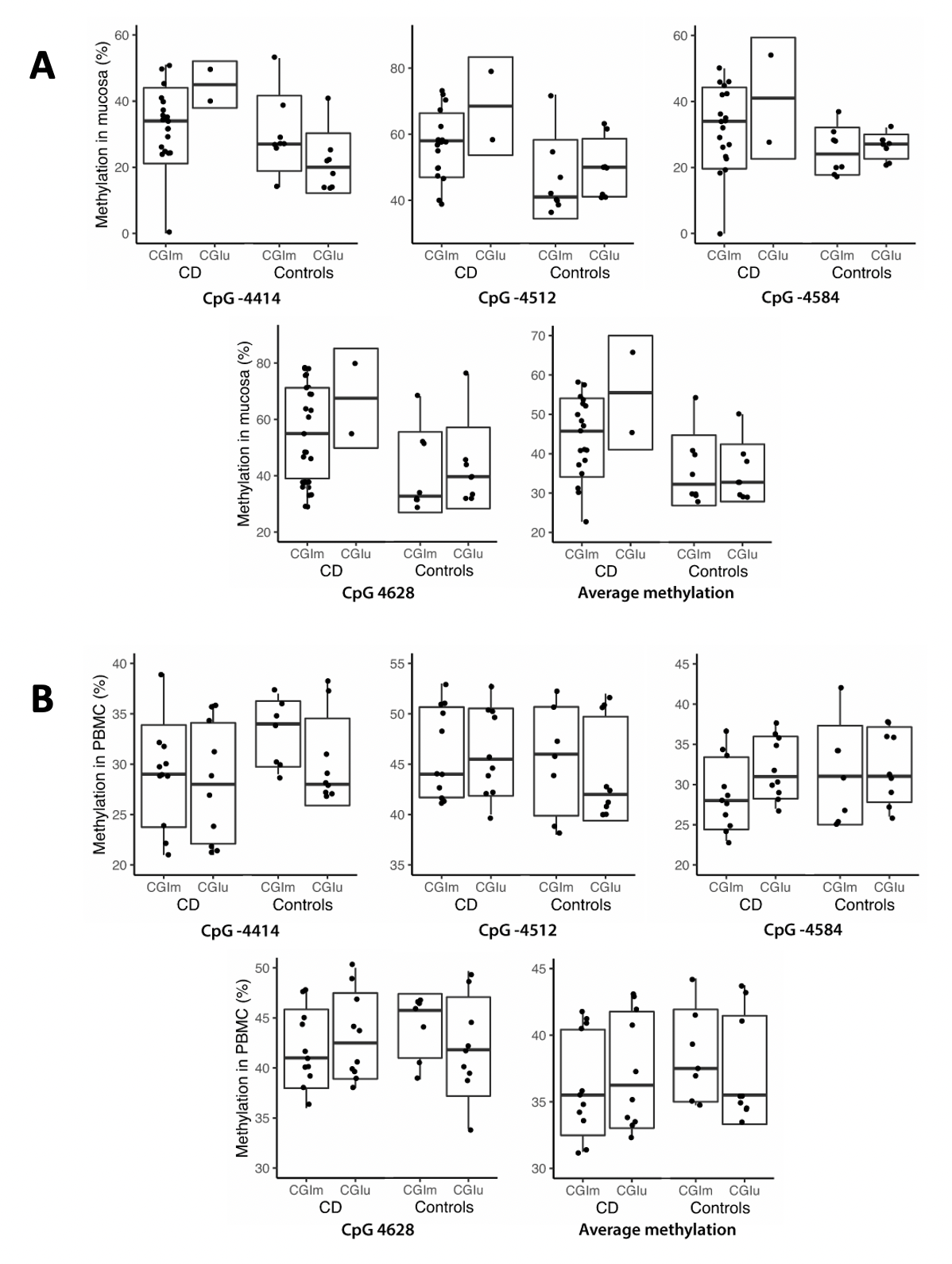


### Additional Tables

### Suppl Table S1: Complete individual methylation data in the studied CD patients and controls.

| **Patients** | **Samples** | **CGI**  Methylated (M)  Unmethylated (U) | **CpG Methylation**  **-4414**  (%) | **CpG Methylation**  **-4512**  (%) | **CpG Methylation**  **-4584**  (%) | **CpG Methylation**  **-4628**  (%) |
| --- | --- | --- | --- | --- | --- | --- |
| **Crohn_1** | Mucosa | M | 51 | 58 | 36 | 55 |
|  | PBMC | U | 36 | 50 | 36 | 50 |
| **Crohn _2** | Mucosa | M | 24 | 47 | 34 | 48 |
|  | PBMC | M | 32 | 51 | 34 | 48 |
| **Crohn _3** | Mucosa | M | 45 | 58 | 23 | 38 |
|  | PBMC | M | 29 | 44 | 28 | 36 |
| **Crohn _4** | Mucosa | M | 34 | 58 | 35 | 61 |
|  | PBMC | U | 21 | 42 | 27 | 39 |
| **Crohn _5** | Mucosa | M | 37 | 55 | 27 | 64 |
|  | PBMC | M | 24 | 43 | 25 | 42 |
| **Crohn _6** | Mucosa | M | 24 | 50 | 29 | 46 |
|  | PBMC | U | 29 | 46 | 30 | 44 |
| **Crohn _7** | Mucosa | U | 50 | 79 | 54 | 80 |
|  | PBMC | U | 31 | 50 | 38 | 49 |
| **Crohn _8** | Mucosa | M | 29 | 62 | 42 | 78 |
|  | PBMC | M | 32 | 51 | 37 | 44 |
| **Crohn _9** | Mucosa | M | 0 | 58 | 0 | 33 |
|  | PBMC | M | 29 | 44 | 29 | 40 |
| **Crohn _10** | Mucosa | M | 24 | 39 | 26 | 36 |
|  | PBMC | M | 29 | 42 | 28 | 40 |
| **Crohn _11** | Mucosa | M | 25 | 58 | 19 | 38 |
|  | PBMC | U | 22 | 42 | 30 | 41 |
| **Crohn _12** | Mucosa | M | 35 | 70 | 50 | 63 |
|  | PBMC | M | 30 | 48 | 26 | 39 |
| **Crohn _13** | Mucosa | M | 32 | 50 | 34 | 47 |
|  | PBMC | U | 21 | 40 | 32 | 40 |
| **Crohn _14** | Mucosa | U | 40 | 58 | 28 | 55 |
|  | PBMC | U | 36 | 53 | 36 | 47 |
| **Crohn _15** | Mucosa | M | 36 | 57 | 23 | 48 |
|  | PBMC | U | 24 | 44 | 28 | 38 |
| **Crohn _16** | Mucosa | M | 34 | 40 | 18 | 29 |
|  | PBMC | M | 21 | 41 | 23 | 41 |
| **Crohn _17** | Mucosa | M | 50 | 58 | 32 | 69 |
|  | PBMC | M | 30 | 50 | 34 | 48 |
| **Crohn _18** | Mucosa | M | 32 | 47 | 45 | 69 |
|  | PBMC | M | 39 | 53 | 30 | 45 |
| **Crohn _19** | Mucosa | M | 26 | 72 | 46 | 71 |
|  | PBMC | U | 27 | 45 | 29 | 40 |
| **Crohn _20** | Mucosa | M | 41 | 67 | 46 | 76 |
|  | PBMC | U | 34 | 50 | 35 | 44 |
| **Crohn _21** | Mucosa | M | 40 | 73 | 42 | 78 |
|  | PBMC | M | 22 | 41 | 24 | 38 |
| **Control_1** | Mucosa | U | 25 | 63 | 26 | 46 |
|  | PBMC | U | 37 | 51 | 38 | 49 |
| **Control_2** | Mucosa | U | 14 | 42 | 28 | 34 |
|  | PBMC | U | 27 | 42 | 29 | 40 |
| **Control_3** | Mucosa | M | 14 | 50 | 27 | 40 |
|  | PBMC | M | 29 | 44 | 34 | 41 |
| **Control_4** | Mucosa | U | 22 | 41 | 21 | 32 |
|  | PBMC | U | 28 | 41 | 31 | 34 |
| **Control_5** | Mucosa | U | 27 | 40 | 20 | 32 |
|  | PBMC | U | 30 | 46 | 34 | 47 |
| **Control_6** | Mucosa | M | 26 | 39 | 17 | 29 |
|  | PBMC | M | 29 | 40 | 27 | 42 |
| **Control_7** | Mucosa | U | 18 | 62 | 28 | 44 |
|  | PBMC | U | 38 | 52 | 38 | 45 |
| **Control_8** | Mucosa | M | 14 | 42 | 28 | 34 |
|  | PBMC | M | 37 | 51 | 31 | 47 |
| **Control_9** | Mucosa | U | 14 | 50 | 27 | 40 |
|  | PBMC | U | 27 | 51 | 36 | 50 |
| **Control_10** | Mucosa | U | 22 | 41 | 21 | 32 |
|  | PBMC | U | 28 | 43 | 31 | 40 |
| **Control_11** | Mucosa | M | 27 | 40 | 20 | 32 |
|  | PBMC | M | 35 | 38 | 27 | 39 |
| **Control_12** | Mucosa | M | 29 | 47 | 31 | 52 |
|  | PBMC | U | 31 | 41 | 26 | 42 |
| **Control_13** | Mucosa | M | 53 | 36 | 18 | 32 |
|  | PBMC | M | 34 | 47 | 25 | 44 |
| **Control_14** | Mucosa | M | 27 | 55 | 28 | 53 |
|  | PBMC | M | 30 | 39 | 25 | 46 |
| **Control_15** | Mucosa | U | 41 | 50 | 32 | 77 |
|  | PBMC | U | 27 | 40 | 36 | 39 |
| **Control_16** | Mucosa | M | 39 | 72 | 37 | 69 |
|  | PBMC | M | 36 | 52 | 42 | 47 |

Suppl Table S2: Methylation in mucosa does not depend on rs16260 (C>A) genotype. However, we observed non-significant trend for increased CGI methylation in patients with CC genotype. No had AA genotype was present. Mean (±SD); decimal values rounded to the nearest integer.

|  | AC | CC |
| --- | --- | --- |
| CD patients:   - Methylated CpG island (N) - Average methylation of enhancer CpGs (%) | n=6  4  46 ± 11 | n=15  15  45 ± 9 |
| Control patients*:   - Methylated CpG island (N) - Average methylation of enhancer CpGs (%) | n=7  4  32 ± 10 | n=9  7  39 ± 8 |
